# Health-related quality of life in patients with paediatric inflammatory bowel disease: IMPACT-III validation in Germany

**DOI:** 10.1186/s12955-025-02437-0

**Published:** 2025-10-02

**Authors:** Aletta Boerkoel, Maresa Buchholz, Luisa Tischler, Niklas Weber, Wolfgang Hoffmann, Jan de Laffolie, Neeltje van den Berg, CEDATA Study Group

**Affiliations:** 1 Institute for Community Medicine, University Medicine Greifswald, Ellernholzstrasse 1/2, DE-17487 Greifswald, Germany; 2 German Center for Child and Adolescent Health, Site Greifswald/Rostock, Greifswald, Germany; 3 German Center for Neurodegenerative Diseases, Site Greifswald/Rostock, Greifswald, Germany; 4 Department of General Paediatrics and Neonatology, University Children’s Hospital, University Giessen, Giessen, Germany

**Keywords:** Health related quality of life, Paediatric inflammatory bowel diseases, Psychometric testing, Patient reported outcomes

## Abstract

**Background:**

Health related quality of life (HRQoL) in paediatric patients with inflammatory bowel disease (IBD) is typically measured using the patient reported outcome measure IMPACT-III. This measure has not yet been validated for German patients using the new 4-domain structure. As Germany has a comparatively high prevalence of paediatric IBD and as the IMPACT-III is the main HRQoL outcome measure in use, a validation in the German population is overdue.

**Objective:**

To validate the main patient reported outcome measure of health-related quality of life (HRQoL) in paediatric patients with inflammatory bowel disease (IBD) IMPACT-III in a German patient sample.

**Methods:**

Clinical and HRQoL data was gathered in the CEDATA-GPGE registry. To determine the psychometric performance of the IMPACT-III in a German sample, distribution properties, reliability (Cronbach’s alpha) and validity (correlations with clinical values; known-groups validity by age, sex, and self-rated health) were calculated. In addition, a confirmatory factor analysis was performed to determine the appropriateness of the factor structure.

**Results:**

The IMPACT-III was filled out by 221 patients (Female 46%; *M*_*age*_=14.05; Morbus Crohn *n* = 126; Ulcerative Colitis *n* = 79; unclassified IBD *n* = 18). The total score ranged from 19.29 to 95.00, without the occurrence of ceiling or floor effects. Internal consistency using Cronbach’s alpha was excellent (α = 0.91) for the total scale. The total score correlated strongly with the subscales of wellbeing (*r* = 0.90) and social functioning (*r* = 0.80). Concerning validity, the subscale of wellbeing correlated with self-reported health and clinical assessments. Younger patients (< 14) reported a significantly better HRQoL than older patients (14–17). The 4-domain structure of IMPACT-III could not be confirmed through factor analysis.

**Conclusions:**

The German version of the IMPACT-III is a valid and reliable instrument to measure HRQoL in paediatric patients with IBD. The subscales of well-being and social functioning explain most of the total score. To interpret the subdomains of the IMPACT-III further research in a longitudinal design needs to be done, especially with age-related phrasing of the items.

**Trial registration:**

German Clinical Trials Register DRKS00015505. Registered on 22.01.2019 Inclusion of patients 01.03.2019; https://drks.de/search/en/trial/DRKS00015505.

**Supplementary Information:**

The online version contains supplementary material available at 10.1186/s12955-025-02437-0.

## Introduction

Paediatric inflammatory bowel disease (PIBD), which includes Crohn’s disease (CD), ulcerative colitis (UC) and Inflammatory Bowel Disease Unclassified (IBD U) is a chronic, relapsing condition driven by an immune system dysfunction, with a prevalence in Europe ranging from 31 to 75 per 100.000 under 18-year old persons depending on the European region (southern Europe has lower prevalences than north-western Europe) [1]. Typical symptoms such as diarrhoea, blood in the stool, weight loss, reduced general well-being, nausea and vomiting, abdominal pain and fatigue can dramatically interfere with daily life [2]. This often results in relevant morbidity, long hospital stays, invasive examinations, growth retardation and intake of multiple medications which influence the individuals’ quality of life and of the surrounding family involved [3]. Compared to adults, children and adolescents often face more severe and more extensive inflammation, persisting symptoms and faster disease progression [4]. In addition to clinical parameters, knowledge about the individuals’ self-perceived health is advantageous to determine a holistic disease picture and provide adequate IBD-related treatment [5].

Health-related quality of life (HRQoL) is of increasing importance in the clinical context when it comes to assessing the impact of certain diseases and the effects of their treatment on patients’ well-being [6]. By operationalising self-perceived health through patient-reported outcome measures (PROM) using appropriate generic or disease-specific questionnaires, it can aid in identifying the need for either prevention or intervention and can compare different treatments or patient groups with each other. HRQoL is lower in children, adolescents and young adults with chronic diseases and for those with mental health problems [7–9]. Assessing HRQoL is particularly relevant for paediatric patients with chronic diseases, as these often coincide with mental health difficulties and lower HRQoL compared to adults, which is often effectively identifiable through self-report [10].

The HRQoL measure IMPACT was the first PIBD-specific measure designed for children and adolescents suffering from IBD [11]. Since then, the original version has been refined in the latest version of the IMPACT-III [12], which has been translated in over 70 languages. Most validation research has been done using the 5-domain version, where various results related to the factor structure were found [13–15]. In response, a 4 domain version is available, which has been used to measure HRQoL, but has not been validated yet [16, 17].

As PIBD prevalence in Germany is among the highest in Europe with 61.7/100.000 patients in 2021 [18], validating the only available disease specific PROM for PIBD in a German patient population is needed. The validation of the IMPACT-III in German is particularly important as it is one of the standard measures for determining HRQoL in paediatric gastroenterological research [19].

### Aims and research questions

The aim of this analysis was to determine the reliability and validity of the IMPACT-III instrument to measure HRQoL in German children and adolescents with IBD.

## Methods

### The CLARA-study and participants

The present analysis used data from the CLARA study – a cluster randomised trial to investigate register-based feedback to clinicians and improve care for paediatric patients with inflammatory bowel disease (German Clinical Trials Register DRKS00015505). The CLARA study aimed to assess the effectiveness of feedback from a clinical registry on guideline-based care [20]. It was performed using the CEDATA-GPGE registry. Paediatric patients (aged 9–17) diagnosed with an IBD were recruited in 47 participating paediatric gastroenterology centres in Germany. Data was collected between 03.2019 and 03.2022. Informed consent was retrieved from all participating legal guardians and their children. The study received ethical approval from the ethics board of the university hospital Gießen (file number 07/11).

### Measurements

During the patient visit, treating physicians worked through a structured online form with parameters including sociodemographic (e.g. age, sex), patient self-reported and clinical (e.g. disease activity, abdominal pain, stool count) variables at baseline. The answers were directly transferred into the CEDATA-GPGE register. In addition to this, the patient was asked to fill out the paper-pencil version of the HRQoL questionnaire IMPACT-III.

#### Self-reported health

*The IMPACT-III Questionnaire* is a self-report measure for children and young people aged 9–17 years with inflammatory bowel diseases. It consists of 35 closed questions which are assigned to the following four main domains: well-being (12 items), emotional functioning (7 items), body image (4 items) and social functioning (11 items). Item 31 (“How often did you have to pass wind in the last 2 weeks?”) is not allocated to a domain [21]. The IMPACT-III uses 5-point Likert scale ranging from 0 to 4 for all items. The linearly transformed outcome score ranges from 0 to 100, with higher scores indicating higher HRQoL.

*Self-rated health (SRH)* was assessed with a one-item verbally presented measure, ranging from “very good” to “very bad”, designed as 5-point Likert Scale.

*Abdominal Pain* was assessed with a one item verbally presented question asking about abdominal pain during the day and night. It was classed by patients at none, mild, moderate or severe pain.

*Limitations in daily activities* was assessed with a one item verbally presented question asking how limited patients feel in their daily lives because of various impairments that people with IBD can experience. It was classed by patients as no limitations, minor limitations, or major limitations.

#### Clinical assessment

##### Current disease activity

Disease activity in IBD describes the degree and intensity of inflammation and symptoms in the digestive tract. Typical symptoms are abdominal pain, diarrhoea, bleeding, fever and general discomfort. Low disease activity often means mild symptoms or an asymptomatic course with low levels of inflammation, while high disease activity is characterized by severe symptoms and significant signs of inflammation. Disease activity often varies in relapses interspersed with periods of remission (symptomless/asymptomatic) [22, 23]. It was classed by medical doctors as remission (inactive), mild activity, moderate activity, severe activity.

##### Extraintestinal symptoms

Extraintestinal symptoms are clinical manifestations that occur outside the gastrointestinal tract and are common in IBD. These symptoms affect other organs and tissues of the body and can occur in different areas. They can occur in parallel with the bowel disease or be independent of the disease activity in the bowel. Typically, they do not impact HRQoL. The following extraintestinal symptoms were identified: eye, liver/gallbladder/pancreas, nephritis, joint: peripheral inflammation, joint: peripheral pain, skin, PSC/overlap, acute pancreatitis, or spine. Due to the heterogeneity of results, the variable was recoded into extraintestinal symptoms present “yes/no”.

### Statistical analysis

To assess the psychometric performance of the IMPACT-III in total as well as with regard to the four domain scores separately, we analysed completeness of data (frequency of missing values), distributional properties, validity (convergent validity, known-groups validity, factor structure) and reliability (internal consistency).

*Completeness of data* as an indicator of acceptance was recorded by the frequency of missing values in the IMPACT III (item-level).

*Distributional properties* were examined on the total and the domain scores as means, standard deviation, range, floor and ceiling effects for the IMPACT-III.

*Validity* To capture the IMPACT-III (convergent) construct, the association between the instrument’s total score and clinical and health-related variables from the registry (specialist indication of disease activity, self-rated health, limitations in daily activities, abdominal pain) was done, using Spearman correlation coefficients with the following interpretation: r_sp_< 0.3 small, 0.3 ≥ r_sp_ <0.5 moderate, and r_sp_≥0.5 high/strong [24]. Additionally, the IMPACT-III’s ability to distinguish between different health states was analysed, using the concept of known-groups. To do so, paired t-tests and one-way ANOVA was used to compare mean responses for the following groups: male vs. female, age groups (median split), CD vs. UC, disease activity (remission to severe). For all comparisons Cohen’s *d* or Cohen’s *f* were calculated, interpreted as follows: 0.20 to < = 0.50, small effect; >0.50 to < = 0.80, medium effect; >0.80, large effect [24]. To determine the construct validity of the IMPACT-III a factor analysis on all items using the methodology of Werner et al. [14] was initially followed and complemented with the detailed description of Uggla et al. [15], to ensure comparability with the literature. Initially a confirmatory factor analysis (cfa) was performed using maximum likelihood estimations to assess the four-domain structure of the IMPACT-III. The model was evaluated using normed chi-square, comparative fit index, standardised root mean square residual and root mean square error of approximation [25]. For the following exploratory factor analysis (efa), a parallel analysis was used to determine the optimal number of factors to retain, after which principal component analysis was conducted. Varimax rotation was used, and items were included in a domain score when factor loadings exceeded 0.5 [14, 15].

*Reliability* We tested the internal consistency of the four IMPACT-III domains, total score and for the proposed factors of the two factor analyses (cfa and efa) using Cronbach’s Alpha to describe the extent to which the items of the respective subscales measure identical/similar characteristics. A Cronbach’s α value > 0.7 was indicated as acceptable [26]. Pearson’s correlation was used to determine the correlations between subscales and Spearman correlation to determine correlation between disease severity and (sub-)scales.

The statistical program packages SAS and R were used for the analysis and visualization of the results. Statistical tests were two tailed with significance at *p* < 0.05. Descriptive statistics were calculated for patient characteristics and IMPACT-III scales.

## Results

### Sample characteristics

The CLARA study included 319 patients at baseline with *n* = 221 patients (MC: *n* = 126; CU: *n* = 79, IND: *n* = 18), who completed the IMPACT-III questionnaire.

The sample characteristics are presented in Table 1. More than half of all patients had CD, followed by UC and IBDU. The mean age at baseline was 14 years (range 9 to 17 years old) and 102 patients (46%) were female. The majority of patients had a moderate disease activity. Patients most frequently reported a moderate SRH; the mean total IMPACT-III score was 66.3.

**Table 1:** Characteristics of the sample at baseline

Sociodemographic and medical variables characteristics	
Female sex, *n* (%)	102 (46)
Age at diagnosis, years, *M* (95% CI)	14.01 (13.69–14.32)
Age at HRQoL assessment, years, *M* (95%CI)	14.05 (13.74–14.36)
IBD Diagnosis, *n* (%)	
Crohn’s disease Ulcerative Colitis Inflammatory Bowel Disease Unclassified	126 (56) 79 (36) 18 (8)
**Disease-related variables**	
Extraintestinal manifestations, *n* (%)	
Yes No Unknown	39 (19) 139 (63) 43 (19)
Current disease activity, *n* (%)	
Inactive Mild Moderate Severe Unknown	30 (14) 62 (28) 92 (42) 33 (15) 4 (5)
**Patient-reported Outcomes**	
IMPACT III total score, *M (95% CI)*	66.60 (64.74–68.47)
Self-rated health, *n* (%)	
Very good Good Moderate Bad Very bad	3 (2) 36 (27) 55 (41) 33 (25) 7 (5)

### Completeness of data

The IMPACT-III was filled out by 221 patients. Thereof, 216 patients answered more than 30 questions, which meant that the questionnaire could be analysed according to the rules set out by Otley et al. [21]. Overall, 188 questionnaires were complete, the remaining 28 questionnaires had informative entries for 30 to 34 items.

### Acceptance

All items had a non-response rate of less than 5%, indicating that there were no acceptance problems with single items. The item with the largest number of non-responses was item 27 “Does IBD make it difficult to travel or to go on holiday?” with *n* = 11 (4.98%). Overall, no problematic non-response items could be identified.

### Distributional properties

Table 2 depicts the findings of the IMPACT-III distributional properties. The total score ranged from 19.3 to 95.0, without the occurrence of ceiling or floor effects. Regarding the sub domains, patients reported the lowest mean score in well-being (one patient) and the highest in social functioning (nine patients), body image (one patient) and wellbeing (one patient).

**Table 2:** Distributional properties of the IMPACT-III, in total and sub domains for *n* = 216

	Mean (95% Confidence Interval)	Minimum -Maximum (range)	Ceiling effect	Floor effect	Skewness	Kurtosis
**Total Score**	66.60 (64.74–68.47)	19.29–95.00 (75.71)	--	--	-0.22	-0.45
**Sub domains**						
Well-being	58.34 (55.42–61.26)	0.00–100.00 (100.00)	0.45%	0.45%	-0.22	-0.73
Emotional functioning	62.34 (60.02–64.67)	14.29–96.43 (82.14)	---	---	-0.30	-0.60
Body Image	64.55 (62.48–66.63)	12.50– 100.00 (87.50)	0.45%		-0.37	0.10
Social functioning	81.63 (79.90–83.35)	34.09–100.00 (65.91)	4.1%	---	-0.68	0.04

### Validity

#### Convergent validity

Convergent validation showed that the wellbeing scale correlates statistically significantly low to moderately with all clinical markers, indicating that with a decrease, SRH and disease activity of the patient also get worse. Similar findings can be reported for the total score (r_sp_<0.3). The correlation is small, but statistically significant, indicating a trend that the total IMPACT-III score responds accordingly to expected clinical outcomes (see Table 3).

**Table 3:** Spearman correlation between the original IMPACT-III sub and total scales and measures of health States

	Self-rated health (*n* = 129)	Current disease activity (*n* = 212)	Activity Limitation (*n* = 129)	Abdominal pain (*n* = 190)
Total score	-0.28**	-0.12	-0.22*	-0.20*
Wellbeing	-0.37***	-0.16*	-0.36***	-0.27**
Emotional functioning	-0.12	-0.01	-0.04	-0.04
Body image	-0.14	-0.17	-0.14	-0.13
Social functioning	-0.14	-0.07	-0.00	-0.06

*p < 0.05 **p < 0.001 ***p < 0.0001

Table 4 presents the IMPACT-III scores in sub-samples of known-groups. Statistically significant lower HRQoL IMPACT-III scores were observed in individuals with higher age (≥ 14 years old), with female sex and increased disease activity. Regarding the current disease activity, no significant group difference was found. No differentiation was found for the type of diagnosis (CD vs. UC) or in the presence or absence of extraintestinal symptoms-.

**Table 4:** Known-groups validation with average score of the total IMPACT-III score. Significance levels for paired t-test and a one-way ANOVA are presented

Sub groups	M (95% CI)	*n*	*p*	d
**Age groups**				
<14 years old	68.97 (66.35–71.60)	94	0.02*	0.31
≥14 years old	64.65 (62.07–67.24)	123		
**Sex**				
Female	64.05 (61.24–66.85)	99	0.01*	0.34
Male	68.77 (66.30-71.23)	117		
**Diagnosis^1**				
Morbus Crohn	66.84 (64.37–69.30)	120	0.64	0.07
Ulceritive Colitis	65.90 (62.67–69.14)	79		
**Current disease activity**			0.17*	-
Inactive	70.05 (65.03–75.07)	30		
Mild	67.00 (63.29–70.70)	59		
Moderate	67.20 (64.41–69.98)	90		
Severe	62.36 (57.04–67.68)	33		
**Extraintestinal symptoms**				
Yes	68.66 (64.52–72.80)	32	0.34	0.06
No	65.94 (63.38–68.50)	132		

*M* = Mean; CI = Confidence Interval ^1 unclassified IBD was excluded

#### Construct validity

The confirmatory factor analysis yielded χ2 /df of 2,04, indicating an acceptable model fit [27]. The comparative fit index was 0.75, which is less than acceptable fit cut-offs (scores above 0.90 are considered acceptable) [28, 29]. The standardised root mean square residual was 0.079 which meets the expectation of being below 0.10 [30], and the root mean square error of approximation was 0.07 which indicates an acceptable model fit [31]. Standardised error variances were significant for all included items. For almost all variables, more than half of the variances was not explained by the factors. Based on the recommendations by Schweizer [25] we can decide the proposed model is not optimally fitting to the data. Thus, a principal component analysis was conducted.

Kaiser’s Measure of Sampling adequacy was sufficient with KMO = 0.84. Bartlett’s test of sphericity indicated that correlations between items were sufficiently large for a PCA (χ2 = 2588.40, df = 595, *p* < 0.0001). A parallel analysis was performed to determine the number of factors to retain. The overlap between the simulated and the traditional scree plot indicated the number of factors to retain. In this case, the lines overlapped at step 3 and step 4. Therefore, an exploratory factor analysis was performed using both 3 and 4 factors. The factor loadings for both the 3 factor and the 4-factor model can be found in the Additional material 1. The identified factors did not include all items of the measure (for the 3-factor model 21 items can be included, for the 4-factor model 22 items), nor are the items sorted to the original factor structure. Rather a qualitatively different summary of items became evident.

### Reliability

Cronbach’s alpha for the total IMPACT-III scale showed excellent reliability with α = 0.91 and the original subscale of wellbeing good reliability (α = 0.89). Acceptable reliability was found for the original subscales of emotional (α = 0.72) and social functioning (α = 0.75). The original subscale of body image showed questionable reliability (α = 0.59). The original impact scales were moderately intercorrelated (*r* = 0.43 to 0.60), but moderately to strongly correlated to the total impact score. The strongest correlations were observed with the original subscale of wellbeing (*r* = 0.90) and social functioning (*r* = 0.80).

## Discussion

The current analysis aimed to determine whether the IMPACT-III is a reliable and valid instrument to measure HRQoL in PIBD in Germany. This is the first study to address the psychometric performance of the IMPACT-III in a German sample of children and adolescents with PIBD. The findings showed that the IMPACT-III is a valid and reliable instrument for measuring HRQoL in this population, but that the recommended subscales might need revising to provide more appropriate qualitative meaning.

The mean total score of the IMPACT-III was similar to that reported in the Malaysian validation [17], but lower than the score in a Saudi-Arabian sample [16]. No studies using the 4-factor structure in countries with similar cultural contexts as in Germany were identified. Compared to both samples, the average score on the subscale of social functioning was higher. As the questionnaire was presented during the patients’ diagnostic visit at the specialist institution, and the patients thus had no or minimal disease management, the worse overall HRQoL can be expected. The IMPACT-III scale had a wider score spread in the German sample compared to a sample from Saudi-Arabia, where the lowest score was 44 points [16]. Other papers did not report scoring spread. The total and subscales did not show floor or ceiling effects.

Score differences were identified between older and younger patients, where patients aged 14 and older reported a worse HRQoL than younger patients. This was also found in the European KIDSCREEN study, where general HRQoL was measured in over 20.000 healthy children and adolescents [32] and in the German BELLA Study, where HRQoL was measured in 1700 German children and adolescents [9]. Because of this similar trend in a healthy population, the decline in HRQoL cannot necessarily be related to a worsening in the disease. This differentiation can be determined by presenting a generic HRQoL measure in addition to the disease specific IMPACT-III. In addition to this, some items of the IMPACT-III might not be appropriate for the younger respondents, such as the item asking after boy- and/or girlfriends, indicating a need for research to develop age-specific HRQoL measures or items across different developmental stages by following established guidelines such as those provided by ISPOR, the professional society for health economics and outcomes research. One of their recommendations for outcomes research with children and adolescents is to consider age limits: the age range from eight to eleven and from twelve to eighteen, respectively, which differs from the use of the IMPACT-III for children between the ages of 9 and 17 [33].

A significant difference between mean scores for males and females was found, females scored lower than males. This finding fits the general population findings, but also the IMPACT-III results from Saudi-Arabia [16] and Malaysia [17]. Our findings were similar to the average scores obtained from disease activity, and diagnosis groups.

Cronbach’s Alpha values show a good reliability of the total scale, but the subscales range from acceptable to questionable reliability. Our findings are slightly worse for the subscales compared to the original 4-domain validation paper [12]. Few studies have been published using the proposed 4-domain structure, thus limiting our ability to compare our findings. One study out of Malaysia reported an overall α = 0.87, which is a slightly lower score than our α = 0.91 [17]. The wellbeing scale correlated the most with the total score, implying that the total score of the IMPACT-III is driven by scores on that subscale. This was supported by the convergent validation with the registry documented clinical finding of patient self-reported wellbeing, which was significantly correlated to the IMPACT-III total score and to the subscale of wellbeing. In fact, all clinical markers correlated statistically significantly, but numerically weakly with the subscale of wellbeing and the total score, but not with the other three subscales. This finding was surprising, as it is known that especially social and emotional functioning is negatively influenced by IBD [10, 34].

Following the methodology of Uggla et al. [15] and Werner et al. [14], a confirmatory factor analysis was run. This showed that the original factor structure did not fit the data well. The following principal components analysis showed that either a 3- or a 4-factor structure would best fit the data. However, as the present study was not conceived as a validation study, no recommendations for the factor structure for the IMPACT-III DE can be reliably derived. Our findings imply that the IMPACT-III in its current state and using the recommended factor structure, while workable and usable in principle, might not be best fitting for a German patient population.

### Strengths and limitations

Compared to other studies validating the IMPACT-III, the current study has a large sample which reflects the German PIBD population [18].

This study was not designed as a validation study, explaining why further important psychometric properties like test-retest reliability and responsiveness could not be evaluated. As part of a cluster-randomised trial, HRQoL data and clinical data was collected at first presentation when the RCT started. One limitation of this approach was that at the time of recruitment the patient population had only recently been diagnosed. This could have limited the reliability of the IMPACT-III, as at this early time point some items (e.g., How has your IBD affected your family?) might not be fully understood by the patients yet. This is why our factor analysis should not be over-interpreted. With increasing illness familiarity items might be understood better and could thus be allocated to factors more appropriately. Similarly, no generic HRQoL measure was available to compare the results of the IMPACT-III with. Another limitation was that, unlike in most previously published literature, we were unable to calculate the pcdai/pucai scores, as not all needed variables were collected for the patient registry. As proxy of this, the clinical assessment of the physician was used. For this assessment however, it should be off note that inter-observer variability could not be accounted for. The clinical assessment is part of physician training and should follow similar diagnostic guidelines. The large number of participating centres prohibits any further statistical analysis on the sample, as the subgroups are too small to compare. The missing patients that were included in the RCT but that did not fill out the questionnaire could indicate participation bias, however no differences between these groups on sex, age, diagnosis or recruitment site could be identified.

Considering these limitations, the current findings can support clinicians and researchers working with German PIBD-patients, that the overall scale gives a good indication of HRQoL for their patients/participants and can be used in practice.

## Conclusion

The IMPACT-III is a valid and reliable instrument to measure HRQoL in German children and adolescents with IBD. The subscales need further scrutiny to improve their ability to qualitatively explain HRQoL subdomains.

## Supplementary Information

Below is the link to the electronic supplementary material.

Supplementary Material 1

## Data Availability

The datasets used and/or analysed during the current study are available from the corresponding author on reasonable request.
